# cGAS-STING Signaling Pathway and Liver Disease: From Basic Research to Clinical Practice

**DOI:** 10.3389/fphar.2021.719644

**Published:** 2021-08-18

**Authors:** Bangjie Chen, Xianyue Rao, Xinyi Wang, Zhipan Luo, Jianpeng Wang, Shuyan Sheng, Yuchen Liu, Ning Zhang, Shiyu Jin, Haosong Chen, Chenyu Sun, Tao Xu, Yingying Du

**Affiliations:** ^1^ The First Affiliated Hospital of Anhui Medical University, Hefei, China; ^2^ First Clinical Medical College, Anhui Medical University, Heifei, China; ^3^ AMITA Health Saint Joseph Hospital Chicago, IL, United States; ^4^ Inflammation and Immune Mediated Diseases Laboratory of Anhui Province, Hefei, China; ^5^ School of Pharmacy, Anhui Medical University, Hefei, China

**Keywords:** CGAS, STING, liver, inflammation, cancer

## Abstract

The cGAS-STING signaling pathway is an autoimmune inflammatory pathway that can trigger the expression of a series of inflammatory factors represented by type 1 interferon. Recent studies have found that the cGAS-STING signaling pathway played a significant role in liver physiology and was closely related to the progress of liver diseases. For example, activating the cGAS-STING signaling pathway could significantly inhibit hepatitis B virus (HBV) replication in vivo. Moreover, the cGAS-STING signaling pathway was also closely associated with tumor immunity in hepatocellular carcinoma (HCC). This review summarized the role of the cGAS-STING signaling pathway in several common liver diseases, especially the current application of the cGAS-STING signaling pathway in liver disease treatment, and prospected its future research, which provided a new idea for understanding and treating liver diseases.

## Introduction

Liver disease is a major cause of illness and death, affecting many people worldwide. Liver diseases mainly include viral hepatitis, alcoholic liver disease (ALD), non-alcoholic fatty liver disease (NAFLD), and related liver cirrhosis, liver failure (LF), and hepatocellular carcinoma (HCC). Liver disease is prevalent worldwide with a poor long-term clinical prognosis, leading to severe public health problems and potentially threatening a large part of the global population.

Cyclic guanosine monophosphate–adenosine monophosphate (GMP-AMP) synthase (cGAS), considered as a pattern recognition receptor (PRR) and direct cytoplasmic dsDNA sensor, was discovered by Dr Chen and his group in 2013. When cGAS binds to dsDNA, the cGAS-STING signaling pathway is activated and then induces the expression of type I IFNs and other inflammatory cytokines, triggering innate immune responses (Barber, 2015). As STING is widely expressed in various cell types and can regulate different pathways of programmed cell death, a deeper understanding of the cGAS–STING signaling pathway may bring a new dawn to treat infections, chronic inflammatory diseases, and even cancer.

Recently, researchers found that the cGAS–STING signaling pathway was closely related to the occurrence and development of multiple liver diseases. Therefore, this review summarized the regulatory functions and mechanisms of the cGAS–STING signaling pathway in various liver diseases. Based on this, the therapeutic value of this signaling pathway in liver diseases was also discussed.

## Overview of Liver Disease

Liver diseases include viral hepatitis, ALD, NAFLD, and related liver cirrhosis, LF, and HCC. Liver disease has a wide range of effects all over the world. About 2 million deaths occur each year, of which 1.4 million dies of liver cirrhosis and 0.6 million dies of viral hepatitis and HCC. Liver fibrosis and HCC are the 11th and 16th world death causes, respectively. Taken together, they account for 3.5% of total global deaths.

Furthermore, these statistics are not always accurate because specific death statistics are sparse in many regions, such as Africa. Meanwhile, accurate mortality data are not available for approximately one-third of countries worldwide (Asrani et al., 2019). Therefore, deaths caused by liver disease may exceed estimates.

Frequently, liver diseases are caused by cellular oxidative stress and inflammatory response, leading to excessive extracellular matrix (ECM) production. ICM collagen-dominated ECM deposition destroys the liver’s normal structure, leading to liver dysfunction, followed by fibrosis, cirrhosis, and hepatocellular carcinoma. Specifically, following the onset of hepatocyte injury, cytokines and proteins rapidly increase and initiate inflammatory responses (Jayaraman et al., 2012; Hernández-Aquino and Muriel, 2018). The development of liver disease is a gradual process and irreversible pathological changes that cause great clinical treatment challenges. At present, the clinical treatment of liver disease is mainly drug therapy. The surgical method is only suitable for early-stage HCC but with poor efficacy and a high recurrence rate. Liver transplantation is the only effective treatment for end-stage liver disease, but the lack of a liver source and the adverse effects of lifelong medication limit its application. Fortunately, researchers found that cGAS activated downstream STING proteins and caused a series of downstream reactions, which played a significant role in protecting hepatocytes and provided a new direction of research to treat liver diseases.

## Overview of cGAS-STING Signaling Pathway

cGAS has been identified as a cytosolic pattern recognition receptor and a cytoplasmic DNA sensor that can activate the IFN pathway. cGAS is activated by mislocalized DNA from different sources in the cytoplasm, such as viruses, bacteria, protozoa, mitochondria, and self-DNA from tumors or dead cells (Zheng et al., 2020). Nuclear damage leads to DNA accumulation in the cytoplasm, mainly in the form of a micronucleus. Micronuclei, also known as satellite nucleus, is a kind of abnormal structure containing DNA in eukaryotic cells and a form of chromosome aberration in interphase cells (Fenech et al., 2011). One consequence of micronuclei disruption is that chromosomal DNA can bind to cGAS (Li and Chen, 2018). The C-terminal lobe of cGAS contains a conserved zinc ion-binding region, which mediates its binding to DNA and dimerization of cGAS (Ouyang et al., 2012; Zhang et al., 2020). Meanwhile, the ligands of DNA promote the activation of cGAS mainly by inducing conformational changes around catalytic sites. Moreover, in the DNA binding structure of cGAS, the conformation of the GS-containing loop changes to maintain its stability, which is a major mechanism of cGAS activation by DNA (Donovan et al., 2013; Zheng et al., 2020).

**FIGURE 1:**
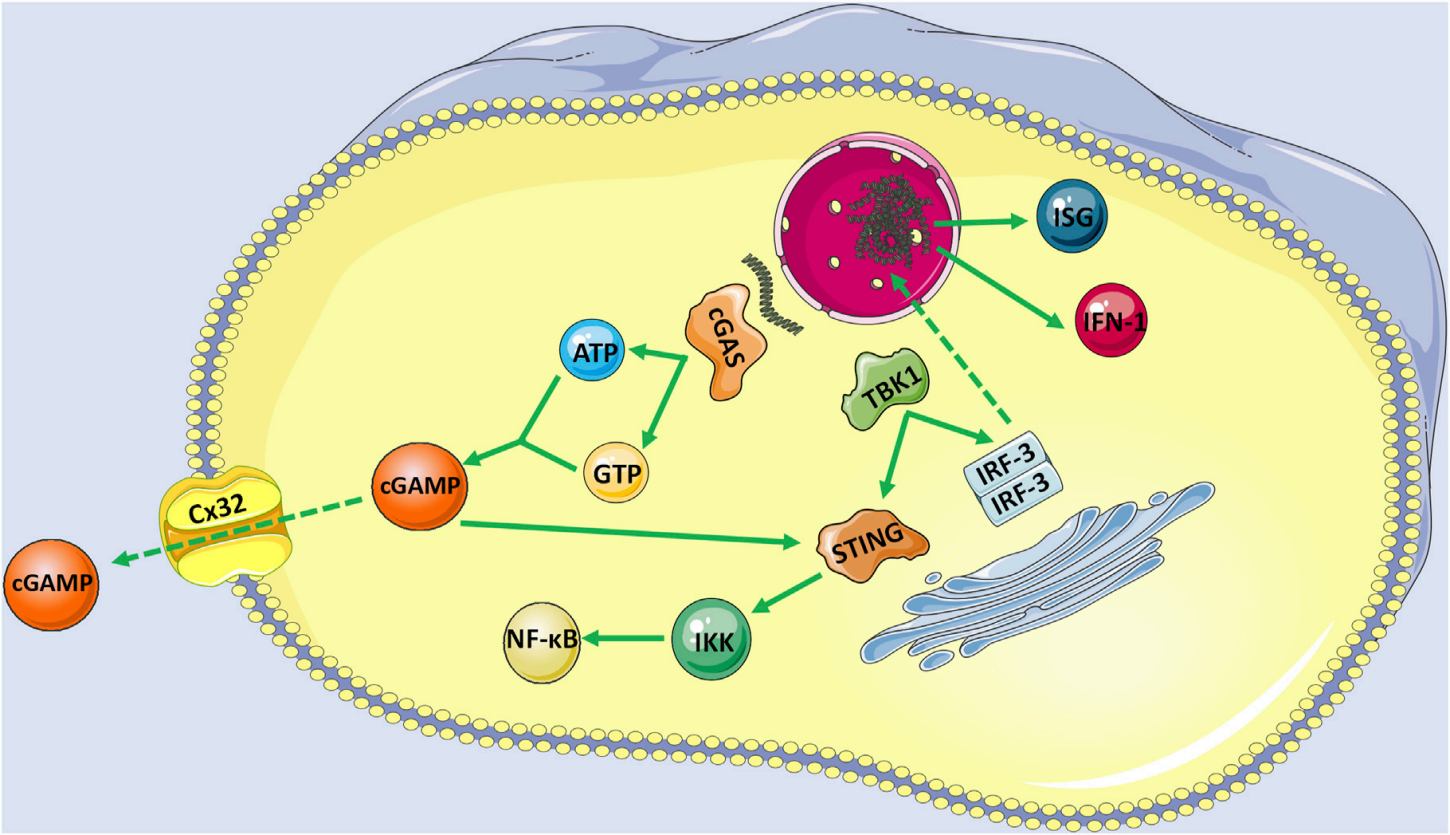
The activation of cGAS-STING signaling pathway with endogenous DNA as an example. When cGAS bind to DNA, ATP and GTP act as substrates to activate cGAS to catalyze the formation of cGAMP, which is a second messenger to activate STING. cGAMP also transactivates STING in adjacent cells with the help of gap junction proteins such as Cx32. Activated STING is firstly palmitoylated in the Golgi apparatus and then recruits the TBK1. Conversely, the C-terminal domain of STING is phosphorylated by TBK1, and phosphorylated STING recruits RF3, which is also phosphorylated and dimerizes by TBK1. Finally, dimerized IRF3 enters the nucleus and plays its role in the transcription of IFN-1 and ISGs. Moreover, STING can also bind to IKK and activate its mediated NF-κB.

STING is a small protein (∼40 KD) located in the endoplasmic reticulum (ER) with four possible transmembrane domains (Ng et al., 2018; Motwani et al., 2019). Normally, STING is retained in the ER by interacting with the Ca^2+^ sensor stromal interaction molecule 1 (STIM1) in the resting state (Srikanth et al., 2019). When cGAS binds to DNA, ATP and GTP act as substrates to activate cGAS and then catalyze the formation of cGAMP, which is a second messenger to activate STING (Murthy et al., 2020). cGAMP also transactivates STING in adjacent cells through gap junction proteins such as Cx32 (Pepin et al., 2020). Activated STING is first palmitoylated at two cysteine residues (Cys88 and Cys91) in the Golgi apparatus and then recruits the kinase TANK-binding kinase 1 (TBK1). Conversely, the C-terminal domain of STING is phosphorylated by TBK1. Phosphorylated STING recruits interferon regulatory factor 3 (IRF3), phosphorylated, and dimerizes by TBK1. Finally, dimerized IRF3 enters the nucleus and plays its role in the transcription of type I IFNs, and interferon-stimulated genes (ISGs) (Mukai et al., 2016; Tao et al., 2016). As we know, IFN is essential for the antitumor immune response mediated by DC and CD8^+^T cells and leads to adaptive immunity (Zheng et al., 2020). Concurrently, STING can also bind to IκB kinase (IKK) and activate nuclear factor-κB (NF-κB), which drives the production of inflammatory genes. Upon the termination of signal transduction, STING is transferred to endolysosomes for degradation (Ma et al., 2015) (Figure 1).

Besides triggering inflammatory storms, the cGAS-STING signaling pathway is also closely associated with apoptosis. Apoptosis is a kind of programmed death mediated by caspase. Apoptotic bodies are engulfed by adjacent cells or macrophages shortly after their formation, thus preventing the activation of inflammatory pathways. It is found that caspase-3 negatively regulated the cGAS-STING signaling pathway, resulting in the separation of IRF3 and STING. Correspondingly, the cGAS-STING signaling pathway was constitutively activated in caspase-9 deficient cells (Rongvaux et al., 2014). On the contrary, the activation of STING also induced ER stress response, which led to apoptosis by activating BAK/BAX and releasing cytochrome C (Cui et al., 2016). Therefore, apoptosis can inhibit the inflammatory response mediated by the cGAS-STING signaling pathway, while ER stress associated with STING activation can trigger apoptosis (Murthy et al., 2020).

In recent years, more evidence shows that the autophagy mechanism is associated with the cGAS-STING signaling pathway. Autophagy involves complex mechanisms, packages damaged or long-lived organelles and proteins in double-membrane vesicles called the autophagosome and transfer them to lysosomes for degradation and recovery (Galluzzi et al., 2017). Usually, autophagy is considered a non-selective process. A few selective autophagy is mediated by ubiquitin-binding proteins (p62, autophagy receptor Optineurin, and NDP52) or proteins with transmembrane domains such as NIP3-like protein X (Nix) (Johansen and Lamark, 2011; Behrends and Fulda, 2012). Studies have shown that cGAS could activate STING-dependent or STING-independent autophagy as host self-protective response, depending on the intensity of the threat of harmful factors and the degree of inflammatory responses required to clear danger signals. In general, autophagy induced by DNA stimulation depends on cGAS and STING. After DNA stimulation, STING protein was colocalized with autophagy marker LC3, while the interaction between cGAS and Beclin-1 promotes autophagy but suppresses the catalytic activity of cGAS and negatively regulates the cGAS-STING signaling pathway to prevent its over-activation. The activation of the STING signaling pathway starts from the transport of STING from the endoplasmic reticulum to Golgi intermediate compartment (ERGIC) and then to Golgi. The membrane transport process of STING is an important step to activate its downstream signaling pathway. In the process of STING transport, cGAMP stimulation can cause the colocalization of STING and ERGIC-53 (ERGIC marker). ERGIC is the main membrane provider in cGAMP induced esterification of LC3 (Gui et al., 2019).

Further experiments show that cGAMP induced autophagy is a new function different from classical autophagy. Surprisingly, researchers found that cGAS induced autophagy protects hepatocytes that lack STING, while STING-mediated autophagy can clear infections effectively (Thomsen et al., 2016). Altogether, cGAS associated autophagic processes play a critical role in liver diseases, which may furnish fresh thought to the treatment for liver disease.

Moreover, recent studies have identified a novel mechanism by which mutant p53 promotes cancer cell survival and evades tumor immune surveillance by suppressing both autonomous and non-autonomous signals (Ghosh et al., 2021). Specifically, by interacting with TBK1, mutant p53 inhibits downstream cGAS-STING cytoplasmic DNA sensing signaling pathway, leading to an inhibited type I IFN response and promoting tumor growth through immune evasion. In general, IRF3, STING, and TBK1 form a trimer complex, a prerequisite for IRF3 activation and downstream signal transduction. This innate immune signaling pathway plays a key role in inhibiting tumor development, leading to immune cell-mediated tumor suppression (Sun et al., 2013; Woo et al., 2014; Dou et al., 2017; Gulen et al., 2017). Meanwhile, TP53 is a critical tumor suppressor gene (Bakhoum and Cantley, 2018), but missense mutations in its DNA binding domain often lead to the loss of its antitumor activity and the production of carcinogenic mutant p53 protein (Mtp53) with functional acquired activity (Eischen, 2016). Mtp53 can block the phosphorylation of TBK1 substrate and the formation of TBK1- STING-IRF3 trimer complex, thus prevent the activation of TBK1 and IRF3 and ultimately leading to the occurrence of immune escape (Ghosh et al., 2021). In conclusion, the cGAS-STING signaling pathway is critically involved in tumor immune escape and has great inspiration for cancer treatment.

In addition to activating immune and inflammatory responses to microbial and self-DNA in the cytoplasm, cGAS can also bind to chromatin, especially after the nuclear membrane breakdown as cells enter mitosis. Recent biochemical studies have shown that cGAS activity was inhibited by two mechanisms during mitosis, N-terminal hyperphosphorylation, and inhibition of oligomerization by chromatin tethering. Both mechanisms prevent the separation of the cGAS phase into liquid droplets, which may benefit the efficient synthesis of cGAMP from cGAS (Li et al., 2021). Taxane drugs are commonly used in cancer chemotherapy because they can stabilize microtubules structure and interfere with the mitotic process (Morris and Fornier, 2008). Studies from the teams of Hironori Funabiki and Christian Zierhut showed that nucleosomes have a higher affinity for cGAS than DNA and inhibit DNA-induced synthesis of cGAMP (Zierhut et al., 2019). When the mitotic process is arrested, cGAS dependent IRF3 phosphorylation will slowly accumulate and promote cell apoptosis through a transcription-independent mechanism. Therefore, it is not difficult to understand that animal experiments and database analysis suggest that cGAS plays a promoting role in paclitaxel chemotherapy. The researchers believe that cGAS, which binds to chromosomes, may not have abnormal results in normal mitosis because mitosis is usually completed in 30 min, while activation of cGAS signals needs to take several hours to occur (Mackenzie et al., 2017).

However, when mitosis is stagnant, even lower cGAS activity can have some functional consequences. As we know, most human somatic cells are diploid, while some specific tissues such as the heart and liver contain polyploid cells, especially liver tissues contain a high proportion of tetraploid, octoploid, and other polyploid cells. The liver is the most important and main detoxification organ in the human body, and toxic substances such as alcohol, hepatitis viruses, or toxic metabolites are prone to induce gene mutations in hepatocytes, whereas polyploidy is thought to be beneficial in providing normal compensatory genes to maintain liver homeostasis. Polyploid cells usually arrest in the middle phase of the cell cycle, the G1 phase. These cells rarely divide and proliferate and eventually progress toward senescence and death. However, after liver injury, polyploid cells will be forced to proliferate and regenerate to repair damaged liver tissue. The division of these polyploid cells is highly susceptible to the generation of aneuploidy, causing the loss of matching chromosomes or chromosomal segments, triggering the amplification of proto-oncogenes or the loss of tumor suppressor genes, ultimately leading to the occurrence of cancer (Zhang et al., 2017). Since cGAS is closely associated with cell mitosis, it may play an important role in liver injury repair and liver cancer progression.

## The Function of the cGAS-STING Signaling Pathway in Liver Disease

### Viral Hepatitis

**FIGURE 2:**
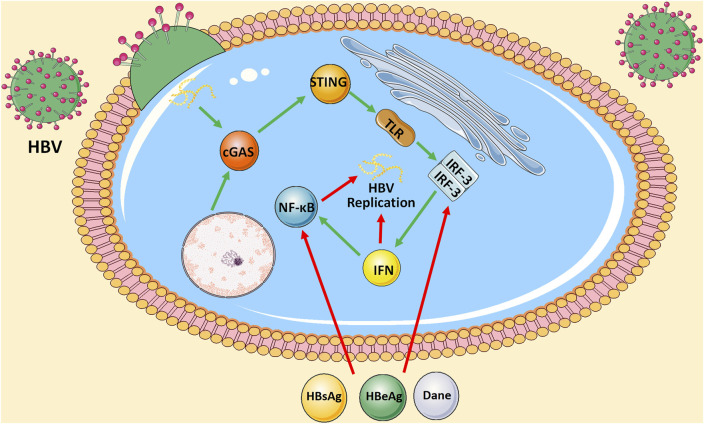
Interaction between cGAS-STING signaling pathway and HBV infection. On the one hand, cGAS-STING signaling either produces IFN by activating downstream TLR3 or further activates NF-κB signaling pathway to inhibit intrahepatic HBV replication. On the other hand, HBsAg, HBeAg, or HBV virions inhibit TLR-induced antiviral activity of hepatocytes by suppressing the activation of IRF-3 and NF-κB.

Viral hepatitis is a major public health problem affecting hundreds of millions of people worldwide with significant morbidity and mortality. Five biologically unrelated hepatotropic viruses account for most of the global burden of viral hepatitis: hepatitis A virus (HAV), hepatitis B virus (HBV), hepatitis C viruses (HCV), hepatitis D virus (HDV), and hepatitis E viruses (HEV). According to the global hepatitis report released in 2017, 1.4 million people died of viral hepatitis infection. Most deaths from viral hepatitis are due to hepatitis B and hepatitis C. It is estimated that 257 million people live with HBV, and 71 million people live with HCV worldwide (WHO, 2017). However, from trends in prevalence, HBV is still the most important pathogenic factor of chronic hepatitis, liver cirrhosis, and HCC. At present, the prevention of HBV is still the main focus. We were unable to clear the virus from patients and HBV carriers or have good control of disease progression during the active phase. Therefore, it is crucial to explore the intracellular molecular mechanisms in HBV infection. Coincidentally, researchers found that HBV infection suppresses cGAS and its effector gene expression in cell culture or mice models (Verrier et al., 2018). Moreover, Hu et al. cotransfected HepG2 cells with HBV replication-competent plasmid (pHBv1.3) and control vectors or plasmids expressing cGAS and STING. IFNβ reporters were cotransfected with plasmids to measure IFN response. It was found that when HepG2 cells were cotransfected with cGAS and STING, the level of HBV RNA was significantly reduced, and the levels of core-associated HBV DNA and secreted HBeAg were reduced by a factor of 5 proportional to the reduction of HBV RNA in cells, which was related to the enhanced activity of IFNβ promoter. Similar results were obtained in the human liver cell line L02 and mouse models dependent on the hydrodynamic injection-based transfection of HBV-replicating plasmid pHBV1.3. On the contrary, the presence of enzyme-inactivated cGAS mutants did not affect HBV replication. Furthermore, researches showed that the knockdown of cGAS in human peripheral blood monocytes (PBMCs) increased the level of intracellular HBV DNA (He et al., 2016). There is an interaction between the cGAS-STING signaling pathway and HBV infection. On the one hand, the cGAS-STING signaling pathway inhibits intrahepatic HBV replication by activating downstream TLR3 to produce IFN-β. On the other hand, HBV evades the perception and antiviral activity of cGAS and its effector pathways through multiple strategies. For example, hepatitis B surface antigen (HBsAg), hepatitis B e antigen (HBeAg), or HBV virions have been reported to inhibit TLR-induced antiviral activity of hepatocytes by suppressing the activation of IRF-3, NF-κB, and extracellular signal-regulated kinase 1/2. Nevertheless, it is worth noting that Kupffer cells, which are an important component of the liver’s innate immune system, may function as a “double-edged sword” in the process of HBV infection. Although it can phagocyte virus-infected cells, the core of the HBV virus can also activate TLR2 on Kupffer cells through the cGAS-STING signaling pathway and inhibit the specific T cell response of HBV by producing IL-10 (Li et al., 2015). In addition, it has been suggested that clearance of apoptotic hepatocytes by Kupffer cells might also play an immunosuppressive role (Sitia et al., 2011). (Figure 2) Collectively, the cGAS-STING signaling pathway provides ideas for the development of novel anti-HBV strategies in the future.

### Alcoholic Liver Disease and Non-Alcoholic Fatty Liver Disease

ALD affects more than 150 million people and remains the leading cause of liver-related mortality worldwide (Schwartz and Reinus, 2012; Luther et al., 2020). In 2010, about 500,000 people died of alcoholic cirrhosis, accounting for 47.9% of all liver cirrhosis-related deaths. Alcohol-related HCC caused another 80,000 death (Rehm and Shield, 2013). The most serious form of ALD is acute alcoholic hepatitis (AH), with a mortality rate as high as 40% within 6 months. The clinical manifestations and pathological changes of ALD range from steatosis (alcoholic fatty liver) to inflammatory and necrotic lesions (alcoholic hepatitis) and progressive fibrosis (cirrhosis) (Cojocariu et al., 2014). The etiology and pathogenesis of ALD are complex, but innate immune dysfunction and excessive inflammatory response have been confirmed to be one of the causes of liver injury in ALD (Nagy, 2015). As mentioned, cGAS triggers innate immune responses by producing the secondary messenger cyclic 2′,3′-cGAMP, which binds to and activates STING, resulting in IRF3 activation. RNA sequence analysis of liver cells from ALD patients showed that the activation level of the cGAS-IRF3 pathway was related to the severity of ALD (Luther et al., 2020). cGAS drives the activation of IRF3 in alcohol-damaged hepatocytes and adjacent parenchyma through gap junctions. Gap junctions are intercellular channels composed of connexin proteins (mainly Cx32), which directly connect the cytosol of adjacent cells and act as cytoplasmic sensors to mediate the rapid transmission of cellular signals (Segretain and Falk, 2004; Luther et al., 2018). In terms of mechanism, gap junctions promote the transfer of 2′,3′-cGAMP from injured to adjacent bystander cells, amplifying the transcellular activation of IRF3 (Ablasser et al., 2013). Activation of IRF3 can lead to alcohol-induced hepatocyte apoptosis, resulting in a strong secondary inflammatory response, which in turn leads to the occurrence of ALD. Therefore, cGAS and Cx32 are the key points in the pathogenesis of ALD and can be used as potential therapeutic targets for liver protection.

Additionally, the rising obesity rate worldwide has also increased liver injury risk caused by NAFLD and non-alcoholic steatohepatitis (NASH) (Altamirano-Barrera et al., 2017). According to the Institute for Health Metrics and Evaluation (IHME), the global prevalence of NAFLD is estimated to be 24%, related to the new epidemic in chronic liver disease. The crosstalk between mammalian target of rapamycin complex 1 (mTORC1) signaling pathway and the cGAS–STING signaling pathway in mammals reveals the important role of this signaling axis in NAFLD (Luo et al., 2018). A more direct association between STING activation and metabolic disorders was shown in NAFLD patients and mice models maintained on a high-fat diet (HFD). The levels of STING were increased. In contrast, the HFD-fed mice without expressing STING (which carried a point mutation in STING T596A leading to no detectable protein) showed a reduction in hepatic steatosis (accumulation of fat in the liver), inflammation, and fibrosis (Papatheodoridi et al., 2020). More specific and in-depth studies have found that STING is less expressed in human and mouse hepatocytes (Luo et al., 2018; Yu et al., 2019). Kupffer cells and monocyte-derived macrophages (MoMF) show high expression of STING. Myeloid-derived STING increases TGF-β1 expression, which in turn induces the activation of hepatic stellate cells (HSCs), ultimately promoting NASH (Luo et al., 2018). In addition, a study of liver samples from 98 patients with NAFLD also showed that STING expression in Kupffer cells and MoMF cells was closely related to that of inflammation and fibrosis (Wang et al., 2020). Therefore, we believe that the cGAS–STING signaling pathway can be a novel and valuable therapeutic target for NAFLD in the future.

### Hepatocellular Carcinoma

**FIGURE 3:**
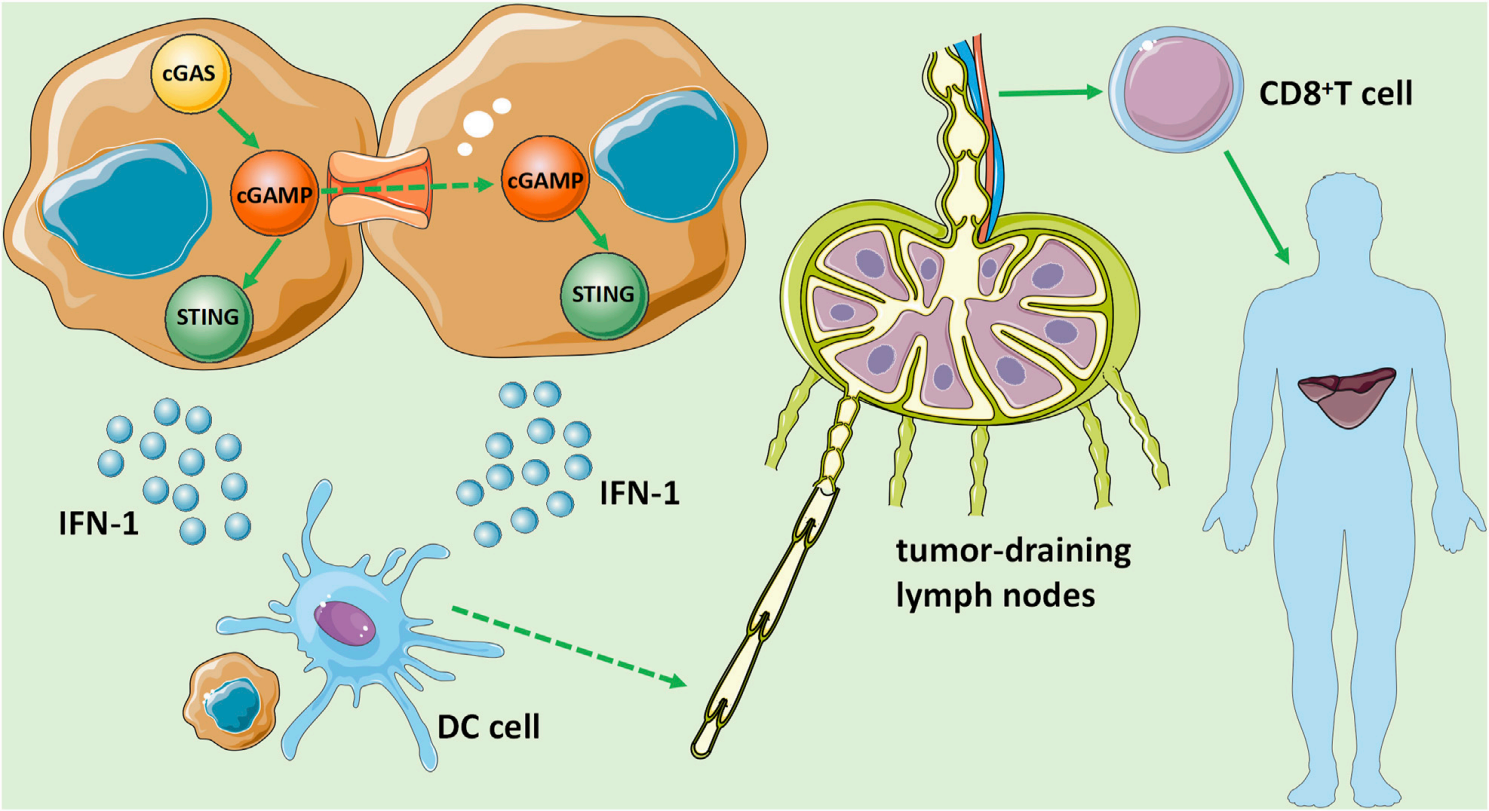
The role of the cGAS-STING signaling pathway in HCC. DNA damage leads to the formation of dsDNA in HCC cells, stimulates and activates the cGAS-STING signaling pathway, and promotes the release of IFN-1, which is critical for DC maturation. Then, DCs migrate to tumor-draining lymph nodes with the help of IFN-1 and cross-activate tumor-specific CD8^+^ T cells, and induce systemic antitumor immunity to control local and distant metastasis of tumor growth.

Primary liver cancer presents a significant health burden as the sixth most common cancer and the fourth leading cause of cancer death worldwide. HCC accounts for 85–90% of all primary liver cancers, which is the most well-studied subtype. Most cases of HCC are associated with cirrhosis, mainly due to chronic HBV or HCV virus infections, followed by other causes such as excessive alcohol consumption and fatty liver disease associated with metabolic syndrome. Although the prognosis of early patients is relatively good with a 5-years survival rate of more than 70%, most HCC patients have developed to advanced stage at the time of diagnosis, resulting in an overall 5-years survival rate of less than 16% (The data comes from IHME). Recently, it is found that the cGAS-STING signaling pathway was involved in the occurrence and development of HCC. In some intense states, cancer cell’s nuclear and mitochondrial DNA is leaky as micronuclei, chromatin fragments, or free telomeric DNA (Woo et al., 2014; Chen et al., 2017; Mackenzie et al., 2017). DNA damage leads to the formation of dsDNA in cancer cells, and upon its stimulation, the cGAS-STING signaling pathway is activated and promotes the release of type I IFN, which is critical for DC maturation (Ranoa et al., 2019). The activation of the cGAS-STING signaling pathway in DCs is a central step of the whole cancer-immunity cycle, which can be initiated by phagocytosis of dead or damaged cancer cells, exosome transfer, and cGAMP gap junctions. Then, DCs migrate to tumor-draining lymph nodes with the help of type I IFNs and cross-activate tumor-specific CD8^+^ T cells, which induce systemic antitumor immunity to control local and distant tumor growth (Papewalis et al., 2008) (Figure 3).

On the other hand, cGAS-STING signaling pathway activation in cancer cells also recruits supportive immune cells to clear carcinoma directly and non spontaneously, such as enhancing the sensitivity of cancer cells to immune attack by natural killer (NK) cells and CTLs. Recently, several novel immunotherapeutic methods have been used in clinical trials for the treatment of HCC, including immune checkpoint inhibitors, new types of immune cell adoption [such as chimeric antigen receptor T cell (CAR-T), TCR gene-modified T cells and stem cells], and microRNAs (Wang and Wang, 2019). However, the effect of these methods is not ideal, but the current findings suggest that STING agonists may be used as adjuvants for cancer vaccines to activate the antitumor immune system and enhance efficacy (Xie et al., 2018).

In addition to several common liver diseases summarized above, the current studies suggest that the cGAS-STING signaling pathway may also involve autoimmune liver diseases. The cGAS-STING signaling pathway can trigger autoimmune diseases by producing type I IFN by recognizing the DNA accumulated in the cytoplasm, and inhibiting the activation of this signaling pathway can slow the progression of diseases.

As mentioned earlier, IFN is essential for mediating acquired immunity and the antitumor immune response. However, cGAS is essential for the expression of IFN-β, as proved by the fact that cGAS knockout mice cannot release type I IFN after detection of cytoplasmic DNA (Li et al., 2013). STING regulates downstream transcription factors of type I IFN in several ways. STING can bind to IKK and activate its mediated NF-κB, which drives the production of inflammatory genes (Ma et al., 2015), thus activating the JAK/STAT pathway in the surrounding cells. NF-κB is a major regulator of inflammation and cell death and is thought to play a central role in liver injury, liver fibrosis, and HCC (Luedde and Schwabe, 2011). The classical IKKβ-dependent NF-κB signaling pathway regulates liver inflammation by controlling the expression of a series of growth factors and cytokines. For example, IL-6 is the most famous in acute inflammation of the liver. It performs many functions by activating STAT3 (He and Karin, 2011). The function of the JAK/STAT signaling pathway in liver cells should not be ignored, including cell proliferation, stem cell maintenance and differentiation, and immune and inflammatory response regulation (Thomas et al., 2015). Studies have shown that it is closely related to liver fibrosis and the occurrence of liver cancer (Mair, 2011), especially STAT3. STAT3 is not only a key anti-inflammatory signal molecule in liver inflammation and fibrosis (Zhao et al., 2021) but also an important factor in promoting the occurrence of liver cancer (Mair, 2011). STING can also activate NF-κB by activating TBK1 through E3 ubiquitin ligase TNF-receptor associated factor 6 (TRAF6)-dependent pathway to activate NF-κB, which translocates to the nucleus to activate the expression of IFN-β (Abe and Barber, 2014). In addition, the frequent necrosis in tumor cells leads to DNA damage and nuclear rupture, and the persistent presence of DNA in the cytoplasm will lead to the chronic activation of cGAS-STING, which has the opposite effect, resulting in the activation of STING-dependent non-normative NF-κB pathway (Reislander et al., 2019). Taken together, STING is considered to be the hub for inducing different downstream transcriptional responses in liver diseases, (Table 1) which not only has the effect of regulating inflammation and antitumor but also can activate these related signal pathways to cause the same results.

**TABLE 1:** Role of cGAS–STING signaling pathway in liver disease and relevant therapeutic target.

Disease	Role of cGAS–STING signaling pathway	Relevant therapeutic targets	Reference(PMID)
Viral hepatitis B	-	TLR3	29679386 (Verrier et al., 2018), 27902332 (He et al., 2016)
Alcoholic liver disease	+	Cx32	30202815 (Luther et al., 2018), 15033576 (Segretain et al., 2004), 24077100 (Ablasser et al., 2013)
Nonalcoholic fatty liver disease	+	mTORC1	30213555 (Luo et al., 2018), 31230380 (Papatheodoridi et al., 2020)
Hepatocellular carcinoma	-	DC cell	30482772 (Ranoa et al., 2019),18209041 (Papewalis et al., 2008), 31352593 (Wang et al., 2019)

a “-” indicates an inhibitory role during disease progression.

b “+” indicates a promoting role during disease progression.

## Advances in Clinical Application

As mentioned above, cGAS-STING signaling pathway activation has a remarkable inhibitory effect on liver viral infection and cancer, thus becoming an exciting target in the field of liver disease immunology and oncology in recent years. Pharmacological modulation of the STING, which is well characterized both structurally and functionally, plays an important role in various liver diseases and is a promising drug target. Currently, the clinical application research of the cGAS-STING signaling pathway is focused on the gene and protein levels.

Advances in therapeutic research at the gene level have focused on hepatitis virus, particularly HBV infection. HBV is like a kind of invisible virus, which cannot trigger any innate immune response. In human hepatocytes, exposure to the naked HBV gene will activate innate antiviral immune responses. However, in the process of HBV infection, it is not detected to a large extent, which may be due to the packaging of DNA in the viral capsid. Another explanation for this lack of sensing is the absence of functional STING protein in hepatocytes. The basal expression of cGAS has the activity of anti-HBV infection, including reducing the cccDNA of the virus.

Moreover, in the study of transfection, the activation of the cGAS-STING signaling pathway could impair HBV replication and assembly of HBV. Given its antiviral function, cGAS is a preferred target to solve the problem of HBV escaping immune responses. HBV can inhibit the expression of cGAS and its related genes such as MB21D1, TMEM17, and TBK1 (Verrier et al., 2018). Furthermore, Western blotting analysis showed that STING was undetectable in hepatocytes but strongly expressed in Kupffer cells (liver macrophages). Correspondingly, the innate DNA sensing pathway was not functional in hepatocytes, whereas it was intact in Kupffer cells (Leong et al., 2015; Thomsen et al., 2016). However, specific expression of STING in living hepatocytes can restore the perception of DNA and protect against HBV infection. For example, *in vivo* transfection of mice hepatocytes resulted in about one-third of cells expressing STING, which was sufficient to detect HBV infection and further reduce the viral load in the organ. Since the virus has not evolved to interfere with DNA sensing actively, liver-directed STING gene transfer combined with cGAMP therapy may be a novel method for treating HBV infection. Although the potent cGAS-STING signaling pathway in hepatocytes may produce harmful results over time, transiently elevating the expression level of this signaling pathway can enable a local antiviral IFN response (Thomsen et al., 2016).

Much focus has been on developing targeted agents for clinical applications at the protein level, including agonists and inhibitors. Accumulating evidence suggests key proteins in the cGAS-STING signaling pathway as potential drug targets, and relevant agonists could fundamentally improve the efficacy of current cancer treatments, including surgery, chemotherapy, radiotherapy, and immunotherapy (Su et al., 2019). As we know, surgery is the most common means of treatment for primary HCC. If the surgery is combined with the activation of the cGAS-STING signaling pathway, local recurrence and distant metastasis can be reduced (Liu et al., 2015). Moreover, as a common chemotherapeutic agent in HCC treatment, paclitaxel can stabilize microtubules and interfere with mitosis. As mentioned above, the current study found that cGAS and IRF3 played important roles in the inhibition of tumor growth by paclitaxel, combining an agonist of the cGAS-STING signaling pathway with paclitaxel could significantly improve the efficacy (Zierhut et al., 2019; Du et al., 2021).

Furthermore, the cGAS-STING signaling pathway is also a critical step in the immune response of HCC to radiotherapy. Exogenous DNA damage induced by radiotherapy can produce cytoplasmic DNA in HCC cells, which enters DCs to activate the cGAS-STING signaling pathway and induce the production of type I interferon. When the cGAS-STING signaling pathway is activated by exogenous cGAMP, it can promote the effect of radiotherapy (Wang et al., 2021). In addition, cGAS-STING signaling pathway agonists can also be combined with immune checkpoint blocker therapy, CAR-T therapy, oncolytic virus therapy, etc. (Waidmann, 2018). Certainly, the most mature clinical application research is STING agonist as adjuvant of cancer vaccine. Appropriate adjuvants play an important role in overcoming immune tolerance and enhancing tumor-specific immunity. Researchers have found that innate immune activation was able to enhance antigen-presenting cell (APC) activation, which provided immunogenicity of tumor-associated antigens (TAAs) (Liu et al., 2015). STINGVAX is the first cancer vaccine designed based on STING, containing cancer cells that can secrete granulocyte-macrophage colony-stimulating factor (GM-CSF) and cyclic dinucleotides (CDNs) (Fu et al., 2015). Compared with the vaccine of single GM-CSF secreting cancer cells, the combination with STINGVAX can enhance the infiltration of T cells in tumor tissue (Du et al., 2021). To summarise, STING agonists have shown promising potential in antitumor immunity. However, emerging evidence suggests that the cGAS-STING signaling pathway has potential roles in promoting carcinogenesis and metastasis, making agonists still face great challenges in clinical application. For example, chronic inflammation has cancer-promoting effects (Liang et al., 2015; Chen et al., 2016; Lemos et al., 2016; Song et al., 2017).

In addition, ER stress and autophagy also serve as barriers to the antitumor effects of STING agonists. They participate in advanced cancer progression by enabling cancer cells to survive under stressful conditions, particularly the ER stress response endowing metastatic cancer cells with the potential for immune evasion (Song et al., 2018). Nevertheless, most cGAS-STING signaling pathway agonists have not yet encountered cancer-promoting effects, as only a few doses of treatment can lead to a burst production of type I IFN, which activates the anti-cancer immune system (Flood et al., 2019). Besides STING agonists, its inhibitors likewise hold pharmacological functions. STING inhibitors play an important role in treating inflammatory diseases and autoimmune diseases, so developing inhibitors that effectively target STING may provide a new idea for treating the autoimmune liver.

In conclusion, the cGAS-STING signaling pathway represents a highly promising direction of research in the field of liver diseases, especially viral liver infections and cancer therapy, both at the genetic and protein levels, which shed new light on the treatment of liver disease.

## Future Expectation

The cGAS-STING signaling pathway was first reported in 2013 by Professor Zhijian Chen’s team. As an important pathway in initiating innate immunity, clinical applications and translational studies of the cGAS-STING signaling pathway have been hotspots in medicine and even life sciences. This section will prospect the future research and application of the cGAS-STING signaling pathway based on the latest research achievements and technologies in related fields.

In recent years, studies showed that the intestinal and hepatobiliary tract exhibited host-specific symbiotic colonization, and the colonized microflora played a key role in the occurrence and development of intestinal and liver diseases. The gut microbiota is essential for maintaining an intact intestinal barrier and controlling the metabolic function of the liver (Adolph et al., 2018). The gut microbiota influences some aspects of early liver disease, such as the evolution of hepatic steatosis, especially in ALD and NAFLD (Moschen et al., 2013). Furthermore, in advanced liver disease, there is also a close relationship between the gut microbiota and cirrhosis and its complications (such as HE) (Acharya et al., 2017). The liver also influences and communicates with microbiota through hepatic mediators, including bile acids and inflammatory signals (Adolph et al., 2018). Therefore, bidirectional crosstalk of the liver microbiome is critical in various liver diseases and may become a therapeutic target. Coincidentally, Canesso et al. found that compared with wild-type (WT) mice, STING-/- mice showed defects in intestinal mucosal protection mechanism, including goblet cells reduction, mucus production, and IgA secretion (Miranda et al., 2019). More importantly, the microbial composition also altered in STING-/- mice, mainly characterized by the decrease of Allobacolum and Bifidobacterium groups, along with the increase of Disulfovibrio bacteria (Canesso et al., 2018). From this, it is natural to speculate that the cGAS-STING signaling pathway is likely to impact liver disease by influencing the gut microbiota composition or altering the function of the intestinal barrier, which enriches our understanding of liver disease and may provide new ideas for our treatment.

In addition, the cGAS-STING signaling pathway can be applied to many cutting-edge clinical therapies as well. As we know, specific blockade of the PD-1/PD-L1 inhibitory pathway is one of the most important strategies for targeted therapy of HCC, and the basis for the implementation of this therapeutic strategy is the infiltration of immune cells in tumor tissues (Waidmann, 2018). As mentioned above, an important function of cGAS-STING signaling pathway activation is to recruit tumor-specific immune cells *via* DCs, and thus we have reason to believe that combining cGAS-STING signaling pathway activators with PD-1/PD-L1 specific blockade will certainly greatly improve the efficacy. Moreover, drug targeted delivery has also become a research hotspot at the intersection of medicine and materials science over the past decade. Scientists have utilized nanocarriers to achieve drug-directed delivery, which could decrease the amount of drug required to improve the therapeutic index, reduce the occurrence of systemic toxicity, prolong drug release time for several days after a single administration, and enhance selective targeting to liver cancer cells (Ashfaq et al., 2017). For example, Wei et al. proposed that dsDNA could be combined with gold nanoparticles as the STING activator (AN), and then AN was co assembled with the chemotherapeutic drug doxorubicin (DOX) onto Mn_3_O_4_ nanoflowers. This nano delivery system could successfully activate cGAS-STING signaling pathway mediated immunotherapy, and the synergistic chemotherapeutic drug exhibited good antitumor efficacy while also exhibiting great performance against distant tumors (Hou et al., 2020).

In conclusion, the cGAS-STING signaling pathway is at the forefront of medical and life science research and closely related to other cutting-edge research results in this field, which certainly represents a promising research direction with a bright future.

## Conclusion

As an autoimmune inflammatory pathway, the cGAS-STING signaling pathway triggers an inflammatory storm associated with autophagy, immune escape, and mitosis. Accumulating evidence has implicated the important role of the cGAS-STING signaling pathway in the pathogenesis of multiple liver diseases. In viral hepatitis B and HCC, the cGAS-STING signaling pathway plays an inhibitory role during disease progression, while in ALD and NAFLD, it plays a promoting role. As a newly discovered pathway, it has broad clinical application prospects. Especially in the treatment of viral hepatitis B and HCC, the activation of this pathway can significantly improve the efficacy of existing treatments. In conclusion, the cGAS-STING signaling pathway is closely associated with liver disease progression and represents a significant potential direction for therapeutic research.

## References

[B1] Abe T. Barber G. N. (2014). Cytosolic-DNA-Mediated, STING-dependent Proinflammatory Gene Induction Necessitates Canonical NF- B Activation through TBK1. J. Virol. 88 (10), 5328–5341. 10.1128/jvi.00037-14 24600004 PMC4019140

[B2] Ablasser A. Schmid-Burgk J. L. Hemmerling I. Horvath G. L. Schmidt T. Latz E. (2013). Cell Intrinsic Immunity Spreads to Bystander Cells via the Intercellular Transfer of cGAMP. Nature 503 (7477), 530–534. 10.1038/nature12640 24077100 PMC4142317

[B3] Acharya C. Sahingur S. E. Bajaj J. S. (2017). Microbiota, Cirrhosis, and the Emerging Oral-Gut-Liver axis. JCI Insight 2 (19). e94416. 10.1172/jci.insight.94416 PMC5841881 28978799

[B4] Adolph T. E. Grander C. Moschen A. R. Tilg H. (2018). Liver-Microbiome Axis in Health and Disease. Trends Immunol. 39 (9), 712–723. 10.1016/j.it.2018.05.002 29843959

[B5] Altamirano-Barrera A. Barranco-Fragoso B. Méndez-Sánchez N. (2017). Management Strategies for Liver Fibrosis. Ann. Hepatol. 16 (1), 48–56. 10.5604/16652681.1226814 28051792

[B6] Ashfaq U. A. Riaz M. Yasmeen E. Yousaf M. Z. (2017). Recent Advances in Nanoparticle-Based Targeted Drug-Delivery Systems against Cancer and Role of Tumor Microenvironment. Crit. Rev. Ther. Drug Carrier Syst. 34 (4), 317–353. 10.1615/critrevtherdrugcarriersyst.2017017845 29199588

[B7] Asrani S. K. Devarbhavi H. Eaton J. Kamath P. S. (2019). Burden of Liver Diseases in the World. J. Hepatol. 70 (1), 151–171. 10.1016/j.jhep.2018.09.014 30266282

[B8] Bakhoum S. F. Cantley L. C. (2018). The Multifaceted Role of Chromosomal Instability in Cancer and its Microenvironment. Cell 174 (6), 1347–1360. 10.1016/j.cell.2018.08.027 30193109 PMC6136429

[B9] Barber G. N. (2015). STING: Infection, Inflammation and Cancer. Nat. Rev. Immunol. 15 (12), 760–770. 10.1038/nri3921 26603901 PMC5004891

[B10] Behrends C. Fulda S. (2012). Receptor Proteins in Selective Autophagy. Int. J. Cel Biol. 2012, 673290. 10.1155/2012/673290 PMC3320096 22536250

[B11] Canesso M. C. C. Lemos L. Neves T. C. Marim F. M. Castro T. B. R. Veloso É. (2018). The Cytosolic Sensor STING Is Required for Intestinal Homeostasis and Control of Inflammation. Mucosal Immunol. 11 (3), 820–834. 10.1038/mi.2017.88 29346345

[B12] Chen Q. Boire A. Jin X. Valiente M. Er E. E. Lopez-Soto A. (2016). Carcinoma-astrocyte gap Junctions Promote Brain Metastasis by cGAMP Transfer. Nature 533 (7604), 493–498. 10.1038/nature18268 27225120 PMC5021195

[B13] Chen Y.-A. Shen Y.-L. Hsia H.-Y. Tiang Y.-P. Sung T.-L. Chen L.-Y. (2017). Extrachromosomal Telomere Repeat DNA Is Linked to ALT Development via cGAS-STING DNA Sensing Pathway. Nat. Struct. Mol. Biol. 24 (12), 1124–1131. 10.1038/nsmb.3498 29106411

[B14] Cojocariu C. E. Trifan A. V. Gîrleanu I. Stanciu C. (2014). Alcoholic Liver Disease-Eepidemiology and Risk Factors. Rev. Med. Chir Soc. Med. Nat. Iasi 118 (4), 910–917. 25581947

[B15] Cui Y. Zhao D. Sreevatsan S. Liu C. Yang W. Song Z. (2016). Mycobacterium Bovis Induces Endoplasmic Reticulum Stress Mediated-Apoptosis by Activating IRF3 in a Murine Macrophage Cell Line. Front Cel Infect Microbiol. 6, 182. 10.3389/fcimb.2016.00182 PMC5149527 28018864

[B16] Donovan J. Dufner M. Korennykh A. (2013). Structural Basis for Cytosolic Double-Stranded RNA Surveillance by Human Oligoadenylate Synthetase 1. Proc. Natl. Acad. Sci. 110 (5), 1652–1657. 10.1073/pnas.1218528110 23319625 PMC3562804

[B17] Dou Z. Ghosh K. Vizioli M. G. Zhu J. Sen P. Wangensteen K. J. (2017). Cytoplasmic Chromatin Triggers Inflammation in Senescence and Cancer. Nature 550 (7676), 402–406. 10.1038/nature24050 28976970 PMC5850938

[B18] Du H. Xu T. Cui M. (2021). cGAS-STING Signaling in Cancer Immunity and Immunotherapy. Biomed. Pharmacother. 133, 110972. 10.1016/j.biopha.2020.110972 33254021

[B19] Eischen C. M. (2016). Genome Stability Requires P53. Cold Spring Harb Perspect. Med. 6 (6), a026096. 10.1101/cshperspect.a026096 27252396 PMC4888814

[B20] Fenech M. Kirsch-Volders M. Natarajan A. T. Surralles J. Crott J. W. Parry J. (2011). Molecular Mechanisms of Micronucleus, Nucleoplasmic Bridge and Nuclear Bud Formation in Mammalian and Human Cells. Mutagenesis 26 (1), 125–132. 10.1093/mutage/geq052 21164193

[B21] Flood B. A. Higgs E. F. Li S. Luke J. J. Gajewski T. F. (2019). STING Pathway Agonism as a Cancer Therapeutic. Immunol. Rev. 290 (1), 24–38. 10.1111/imr.12765 31355488 PMC6814203

[B22] Fu J. Kanne D. B. Leong M. Glickman L. H. McWhirter S. M. Lemmens E. (2015). STING Agonist Formulated Cancer Vaccines Can Cure Established Tumors Resistant to PD-1 Blockade. Sci. Transl. Med. 7 (283), 283ra52. 10.1126/scitranslmed.aaa4306 PMC4504692 25877890

[B23] Galluzzi L. Baehrecke E. H. Ballabio A. Boya P. Bravo‐San Pedro J. M. Cecconi F. (2017). Molecular Definitions of Autophagy and Related Processes. EMBO J. 36 (13), 1811–1836. 10.15252/embj.201796697 28596378 PMC5494474

[B24] Ghosh M. Saha S. Bettke J. Nagar R. Parrales A. Iwakuma T. (2021). Mutant P53 Suppresses Innate Immune Signaling to Promote Tumorigenesis. Cancer Cell 39 (4), 494–508. e5. 10.1016/j.ccell.2021.01.003 33545063 PMC8044023

[B25] Gui X. Yang H. Li T. Tan X. Shi P. Li M. (2019). Autophagy Induction via STING Trafficking Is a Primordial Function of the cGAS Pathway. Nature 567 (7747), 262–266. 10.1038/s41586-019-1006-9 30842662 PMC9417302

[B26] Gulen M. F. Koch U. Haag S. M. Schuler F. Apetoh L. Villunger A. (2017). Signalling Strength Determines Proapoptotic Functions of STING. Nat. Commun. 8 (1), 427. 10.1038/s41467-017-00573-w 28874664 PMC5585373

[B27] He G. Karin M. (2011). NF-κB and STAT3 - Key Players in Liver Inflammation and Cancer. Cell Res. 21 (1), 159–168. 10.1038/cr.2010.183 21187858 PMC3193410

[B28] He J. Hao R. Liu D. Liu X. Wu S. Guo S. (2016). Inhibition of Hepatitis B Virus Replication by Activation of the cGAS-STING Pathway. J. Gen. Virol. 97 (12), 3368–3378. 10.1099/jgv.0.000647 27902332

[B29] Hernández-Aquino E. Muriel P. (2018). Beneficial Effects of Naringenin in Liver Diseases: Molecular Mechanisms. Wjg 24 (16), 1679–1707. 10.3748/wjg.v24.i16.1679 29713125 PMC5922990

[B30] Hou L. Tian C. Yan Y. Zhang L. Zhang H. Zhang Z. (2020). Manganese-Based Nanoactivator Optimizes Cancer Immunotherapy via Enhancing Innate Immunity. ACS Nano 14 (4), 3927–3940. 10.1021/acsnano.9b06111 32298077

[B31] Jayaraman J. Jesudoss V. A. S. Menon V. P. Namasivayam N. (2012). Anti-inflammatory Role of Naringenin in Rats with Ethanol Induced Liver Injury. Toxicol. Mech. Methods 22 (7), 568–576. 10.3109/15376516.2012.707255 22900548

[B32] Johansen T. Lamark T. (2011). Selective Autophagy Mediated by Autophagic Adapter Proteins. Autophagy 7 (3), 279–296. 10.4161/auto.7.3.14487 21189453 PMC3060413

[B33] Lemos H. Mohamed E. Huang L. Ou R. Pacholczyk G. Arbab A. S. (2016). STING Promotes the Growth of Tumors Characterized by Low Antigenicity via Ido Activation. Cancer Res. 76 (8), 2076–2081. 10.1158/0008-5472.can-15-1456 26964621 PMC4873329

[B34] Leong C. R. Oshiumi H. Okamoto M. Azuma M. Takaki H. Matsumoto M. (2015). A MAVS/TICAM-1-independent Interferon-Inducing Pathway Contributes to Regulation of Hepatitis B Virus Replication in the Mouse Hydrodynamic Injection Model. J. Innate Immun. 7 (1), 47–58. 10.1159/000365113 25115498 PMC6951042

[B35] Li M. Sun R. Xu L. Yin W. Chen Y. Zheng X. (2015). Kupffer Cells Support Hepatitis B Virus-Mediated CD8+ T Cell Exhaustion via Hepatitis B Core Antigen-TLR2 Interactions in Mice. J.I. 195 (7), 3100–3109. 10.4049/jimmunol.1500839 26304988

[B36] Li T. Chen Z. J. (2018). The cGAS-cGAMP-STING Pathway Connects DNA Damage to Inflammation, Senescence, and Cancer. J. Exp. Med. 215 (5), 1287–1299. 10.1084/jem.20180139 29622565 PMC5940270

[B37] Li T. Huang T. Du M. Chen X. Du F. Ren J. (2021). Phosphorylation and Chromatin Tethering Prevent cGAS Activation during Mitosis. Science (6535), 371. 10.1126/science.abc5386C PMC8171060 33542149

[B38] Li X.-D. Wu J. Gao D. Wang H. Sun L. Chen Z. J. (2013). Pivotal Roles of cGAS-cGAMP Signaling in Antiviral Defense and Immune Adjuvant Effects. Science 341 (6152), 1390–1394. 10.1126/science.1244040 23989956 PMC3863637

[B39] Liang D. Xiao-Feng H. Guan-Jun D. Er-Ling H. Sheng C. Ting-Ting W. (2015). Activated STING Enhances Tregs Infiltration in the HPV-Related Carcinogenesis of Tongue Squamous Cells via the C-jun/CCL22 Signal. Biochim. Biophys. Acta (Bba) - Mol. Basis Dis. 1852 (11), 2494–2503. 10.1016/j.bbadis.2015.08.011 26303640

[B40] Liu C.-Y. Chen K.-F. Chen P.-J. (2015). Treatment of Liver Cancer. Cold Spring Harb Perspect. Med. 5 (9), a021535. 10.1101/cshperspect.a021535 26187874 PMC4561392

[B41] Luedde T. Schwabe R. F. (2011). NF-κB in the Liver-Linking Injury, Fibrosis and Hepatocellular Carcinoma. Nat. Rev. Gastroenterol. Hepatol. 8 (2), 108–118. 10.1038/nrgastro.2010.213 21293511 PMC3295539

[B42] Luo X. Li H. Ma L. Zhou J. Guo X. Woo S.-L. (2018). Expression of STING Is Increased in Liver Tissues from Patients with NAFLD and Promotes Macrophage-Mediated Hepatic Inflammation and Fibrosis in Mice. Gastroenterology 155 (6), 1971–1984. 10.1053/j.gastro.2018.09.010 30213555 PMC6279491

[B43] Luther J. Gala M. K. Borren N. Masia R. Goodman R. P. Moeller I. H. (2018). Hepatic Connexin 32 Associates with Nonalcoholic Fatty Liver Disease Severity. Hepatol. Commun. 2 (7), 786–797. 10.1002/hep4.1179 30202815 PMC6123534

[B44] Luther J. Khan S. Gala M. K. Kedrin D. Sridharan G. Goodman R. P. (2020). Hepatic gap Junctions Amplify Alcohol Liver Injury by Propagating cGAS-Mediated IRF3 Activation. Proc. Natl. Acad. Sci. USA 117 (21), 11667–11673. 10.1073/pnas.1911870117 32393626 PMC7261084

[B45] Ma Z. Jacobs S. R. West J. A. Stopford C. Zhang Z. Davis Z. (2015). Modulation of the cGAS-STING DNA Sensing Pathway by Gammaherpesviruses. Proc. Natl. Acad. Sci. USA 112 (31), E4306–E4315. 10.1073/pnas.1503831112 26199418 PMC4534226

[B46] Mackenzie K. J. Carroll P. Martin C.-A. Murina O. Fluteau A. Simpson D. J. (2017). cGAS Surveillance of Micronuclei Links Genome Instability to Innate Immunity. Nature 548 (7668), 461–465. 10.1038/nature23449 28738408 PMC5870830

[B47] Mair M. (2011). JAK-STAT Signaling in Hepatic Fibrosis. Front. Biosci. 16, 2794–2811. 10.2741/3886 21622209

[B48] Miranda M. C. G. Oliveira R. P. Torres L. Aguiar S. L. F. Pinheiro‐Rosa N. Lemos L. (2019). Frontline Science: Abnormalities in the Gut Mucosa of Non‐obese Diabetic Mice Precede the Onset of Type 1 Diabetes. J. Leukoc. Biol. 106 (3), 513–529. 10.1002/jlb.3hi0119-024rr 31313381

[B49] Morris P. G. Fornier M. N. (2008). Microtubule Active Agents: beyond the Taxane Frontier. Clin. Cancer Res. 14 (22), 7167–7172. 10.1158/1078-0432.ccr-08-0169 19010832

[B50] Moschen A. R. Kaser S. Tilg H. (2013). Non-alcoholic Steatohepatitis: a Microbiota-Driven Disease. Trends Endocrinol. Metab. 24 (11), 537–545. 10.1016/j.tem.2013.05.009 23827477

[B51] Motwani M. Pesiridis S. Fitzgerald K. A. (2019). DNA Sensing by the cGAS-STING Pathway in Health and Disease. Nat. Rev. Genet. 20 (11), 657–674. 10.1038/s41576-019-0151-1 31358977

[B52] Mukai K. Konno H. Akiba T. Uemura T. Waguri S. Kobayashi T. (2016). Activation of STING Requires Palmitoylation at the Golgi. Nat. Commun. 7, 11932. 10.1038/ncomms11932 27324217 PMC4919521

[B53] Murthy A. M. V. Robinson N. Kumar S. (2020). Crosstalk between cGAS-STING Signaling and Cell Death. Cell Death Differ 27 (11), 2989–3003. 10.1038/s41418-020-00624-8 32948836 PMC7560597

[B54] Nagy L. E. (2015). The Role of Innate Immunity in Alcoholic Liver Disease. Alcohol. Res. 37 (2), 237–250. 26695748 10.35946/arcr.v37.2.08 PMC4590620

[B55] Ng K. W. Marshall E. A. Bell J. C. Lam W. L. (2018). cGAS-STING and Cancer: Dichotomous Roles in Tumor Immunity and Development. Trends Immunol. 39 (1), 44–54. 10.1016/j.it.2017.07.013 28830732

[B56] Ouyang S. Song X. Wang Y. Ru H. Shaw N. Jiang Y. (2012). Structural Analysis of the STING Adaptor Protein Reveals a Hydrophobic Dimer Interface and Mode of Cyclic Di-GMP Binding. Immunity 36 (6), 1073–1086. 10.1016/j.immuni.2012.03.019 22579474 PMC3654694

[B57] Papatheodoridi A. M. Chrysavgis L. Koutsilieris M. Chatzigeorgiou A. (2020). The Role of Senescence in the Development of Nonalcoholic Fatty Liver Disease and Progression to Nonalcoholic Steatohepatitis. Hepatology 71 (1), 363–374. 10.1002/hep.30834 31230380

[B58] Papewalis C. Jacobs B. Wuttke M. Ullrich E. Baehring T. Fenk R. (2008). IFN-α Skews Monocytes into CD56+-Expressing Dendritic Cells with Potent Functional Activities *In Vitro* and *In Vivo* . J. Immunol. 180 (3), 1462–1470. 10.4049/jimmunol.180.3.1462 18209041

[B59] Pepin G. De Nardo D. Rootes C. L. Ullah T. R. Al-Asmari S. S. Balka K. R. (2020). Connexin-Dependent Transfer of cGAMP to Phagocytes Modulates Antiviral Responses. mBio 11 (1), e03187–19. 10.1128/mbio.03187-19 31992625 PMC6989113

[B60] Ranoa D. R. E. Widau R. C. Mallon S. Parekh A. D. Nicolae C. M. Huang X. (2019). STING Promotes Homeostasis via Regulation of Cell Proliferation and Chromosomal Stability. Cancer Res. 79 (7), 1465–1479. 10.1158/0008-5472.can-18-1972 30482772 PMC6445702

[B61] Rehm J. Shield K. D. (2013). Global Alcohol-Attributable Deaths from Cancer, Liver Cirrhosis, and Injury in 2010. Alcohol. Res. 35 (2), 174–183. 24881325 10.35946/arcr.v35.2.07 PMC3908708

[B62] Reislander T. Lombardi E. P. Groelly F. J. Miar A. Porru M. Di Vito S. (2019). BRCA2 Abrogation Triggers Innate Immune Responses Potentiated by Treatment with PARP Inhibitors. Nat. Commun. 10 (1), 3143. 10.1038/s41467-019-11048-5 31316060 PMC6637138

[B63] Rongvaux A. Jackson R. Harman C. C. D. Li T. West A. P. de Zoete M. R. (2014). Apoptotic Caspases Prevent the Induction of Type I Interferons by Mitochondrial DNA. Cell 159 (7), 1563–1577. 10.1016/j.cell.2014.11.037 25525875 PMC4272443

[B64] Schwartz J. M. Reinus J. F. (2012). Prevalence and Natural History of Alcoholic Liver Disease. Clin. Liver Dis. 16 (4), 659–666. 10.1016/j.cld.2012.08.001 23101975

[B65] Segretain D. Falk M. M. (2004). Regulation of Connexin Biosynthesis, Assembly, gap junction Formation, and Removal. Biochim. Biophys. Acta 1662 (1-2), 3–21. 10.1016/j.bbamem.2004.01.007 15033576

[B66] Sitia G. Iannacone M. Aiolfi R. Isogawa M. van Rooijen N. Scozzesi C. (2011). Kupffer Cells Hasten Resolution of Liver Immunopathology in Mouse Models of Viral Hepatitis. Plos Pathog. 7 (6), e1002061. 10.1371/journal.ppat.1002061 21655107 PMC3107209

[B67] Song M. Sandoval T. A. Chae C.-S. Chopra S. Tan C. Rutkowski M. R. (2018). IRE1α-XBP1 Controls T Cell Function in Ovarian Cancer by Regulating Mitochondrial Activity. Nature 562 (7727), 423–428. 10.1038/s41586-018-0597-x 30305738 PMC6237282

[B68] Song S. Peng P. Tang Z. Zhao J. Wu W. Li H. (2017). Decreased Expression of STING Predicts Poor Prognosis in Patients with Gastric Cancer. Sci. Rep. 7, 39858. 10.1038/srep39858 28176788 PMC5296877

[B69] Srikanth S. Woo J. S. Wu B. El-Sherbiny Y. M. Leung J. Chupradit K. (2019). The Ca2+ Sensor STIM1 Regulates the Type I Interferon Response by Retaining the Signaling Adaptor STING at the Endoplasmic Reticulum. Nat. Immunol. 20 (2), 152–162. 10.1038/s41590-018-0287-8 30643259 PMC6340781

[B70] Su T. Zhang Y. Valerie K. Wang X.-Y. Lin S. Zhu G. (2019). STING Activation in Cancer Immunotherapy. Theranostics 9 (25), 7759–7771. 10.7150/thno.37574 31695799 PMC6831454

[B71] Sun L. Wu J. Du F. Chen X. Chen Z. J. (2013). Cyclic GMP-AMP Synthase Is a Cytosolic DNA Sensor that Activates the Type I Interferon Pathway. Science 339 (6121), 786–791. 10.1126/science.1232458 23258413 PMC3863629

[B72] Tao J. Zhou X. Jiang Z. (2016). cGAS-cGAMP-STING: The Three Musketeers of Cytosolic DNA Sensing and Signaling. IUBMB Life 68 (11), 858–870. 10.1002/iub.1566 27706894

[B73] Thomas S. J. Snowden J. A. Zeidler M. P. Danson S. J. (2015). The Role of JAK/STAT Signalling in the Pathogenesis, Prognosis and Treatment of Solid Tumours. Br. J. Cancer 113 (3), 365–371. 10.1038/bjc.2015.233 26151455 PMC4522639

[B74] Thomsen M. K. Nandakumar R. Stadler D. Malo A. Valls R. M. Wang F. (2016). Lack of Immunological DNA Sensing in Hepatocytes Facilitates Hepatitis B Virus Infection. Hepatology 64 (3), 746–759. 10.1002/hep.28685 27312012

[B75] Verrier E. R. Yim S. A. Heydmann L. El Saghire H. Bach C. Turon‐Lagot V. (2018). Hepatitis B Virus Evasion from Cyclic Guanosine Monophosphate-Adenosine Monophosphate Synthase Sensing in Human Hepatocytes. Hepatology 68 (5), 1695–1709. 10.1002/hep.30054 29679386 PMC6195855

[B76] Waidmann O. (2018). Recent Developments with Immunotherapy for Hepatocellular Carcinoma. Expert Opin. Biol. Ther. 18 (8), 905–910. 10.1080/14712598.2018.1499722 29995439

[B77] Wang C. Sun Z. Zhao C. Zhang Z. Wang H. Liu Y. (2021). Maintaining Manganese in Tumor to Activate cGAS-STING Pathway Evokes a Robust Abscopal Anti-tumor Effect. J. Controlled Release 331, 480–490. 10.1016/j.jconrel.2021.01.036 33545219

[B78] Wang L. Wang F.-S. (2019). Clinical Immunology and Immunotherapy for Hepatocellular Carcinoma: Current Progress and Challenges. Hepatol. Int. 13 (5), 521–533. 10.1007/s12072-019-09967-y 31352593

[B79] Wang X. Rao H. Zhao J. Wee A. Li X. Fei R. (2020). STING Expression in Monocyte-Derived Macrophages Is Associated with the Progression of Liver Inflammation and Fibrosis in Patients with Nonalcoholic Fatty Liver Disease. Lab. Invest. 100 (4), 542–552. 10.1038/s41374-019-0342-6 31745210

[B80] WHO (2017). GLOBAL HEPATITIS REPORT.

[B81] Woo S.-R. Fuertes M. B. Corrales L. Spranger S. Furdyna M. J. Leung M. Y. K. (2014). STING-dependent Cytosolic DNA Sensing Mediates Innate Immune Recognition of Immunogenic Tumors. Immunity 41 (5), 830–842. 10.1016/j.immuni.2014.10.017 25517615 PMC4384884

[B82] Xie Y. Xiang Y. Sheng J. Zhang D. Yao X. Yang Y. (2018). Immunotherapy for Hepatocellular Carcinoma: Current Advances and Future Expectations. J. Immunol. Res. 2018, 8740976. 10.1155/2018/8740976 29785403 PMC5896259

[B83] Yu Y. Liu Y. An W. Song J. Zhang Y. Zhao X. (2019). STING-mediated Inflammation in Kupffer Cells Contributes to Progression of Nonalcoholic Steatohepatitis. J. Clin. Invest. 129 (2), 546–555. 10.1172/JCI121842 30561388 PMC6355218

[B84] Zhang S. Chen Q. Liu Q. Li Y. Sun X. Hong L. (2017). Hippo Signaling Suppresses Cell Ploidy and Tumorigenesis through Skp2. Cancer Cell 31 (5), 669–684. e7. 10.1016/j.ccell.2017.04.004 28486106 PMC5863541

[B85] Zhang X. Bai X.-c. Chen Z. J. (2020). Structures and Mechanisms in the cGAS-STING Innate Immunity Pathway. Immunity 53 (1), 43–53. 10.1016/j.immuni.2020.05.013 32668227

[B86] Zhao J. Qi Y.-F. Yu Y.-R. (2021). STAT3: A Key Regulator in Liver Fibrosis. Ann. Hepatol. 21, 100224. 10.1016/j.aohep.2020.06.010 32702499

[B87] Zheng J. Mo J. Zhu T. Zhuo W. Yi Y. Hu S. (2020). Comprehensive Elaboration of the cGAS-STING Signaling axis in Cancer Development and Immunotherapy. Mol. Cancer 19 (1), 133. 10.1186/s12943-020-01250-1 32854711 PMC7450153

[B88] Zierhut C. Yamaguchi N. Paredes M. Luo J.-D. Carroll T. Funabiki H. (2019). The Cytoplasmic DNA Sensor cGAS Promotes Mitotic Cell Death. Cell 178 (2), 302–315. e23. 10.1016/j.cell.2019.05.035 31299200 PMC6693521

